# Claudin-11 in health and disease: implications for myelin disorders, hearing, and fertility

**DOI:** 10.3389/fncel.2023.1344090

**Published:** 2024-01-17

**Authors:** Sophia C. Gjervan, Oguz K. Ozgoren, Alexander Gow, Sylvia Stockler-Ipsiroglu, Mahmoud A. Pouladi

**Affiliations:** ^1^Department of Medical Genetics, Centre for Molecular Medicine and Therapeutics, Djavad Mowafaghian Centre for Brain Health, British Columbia Children's Hospital Research Institute, University of British Columbia, Vancouver, BC, Canada; ^2^Center for Molecular Medicine and Genetics, Wayne State University School of Medicine, Detroit, MI, United States; ^3^Department of Pediatrics, The University of British Columbia and BC Children's Hospital, Vancouver, BC, Canada; ^4^Division of Biochemical Genetics, The University of British Columbia and BC Children's Hospital, Vancouver, BC, Canada

**Keywords:** claudin-11, tight junctions, myelin, leukodystrophy, HLD22, hearing, fertility

## Abstract

Claudin-11 plays a critical role in multiple physiological processes, including myelination, auditory function, and spermatogenesis. Recently, stop-loss mutations in *CLDN11* have been identified as a novel cause of hypomyelinating leukodystrophy (HLD22). Understanding the multifaceted roles of claudin-11 and the potential pathogenic mechanisms in HLD22 is crucial for devising targeted therapeutic strategies. This review outlines the biological roles of claudin-11 and the implications of claudin-11 loss in the context of the *Cldn11* null mouse model. Additionally, HLD22 and proposed pathogenic mechanisms, such as endoplasmic reticulum stress, will be discussed.

## 1 Introduction

The myelin sheath, a membrane critical for normal nerve conduction in the central nervous system (CNS), insulates axons and facilitates efficient transduction via depolarization at the nodes of Ranvier (Harry and Toews, 1998). Several diseases associated with myelin abnormalities often have a genetic basis, such as leukodystrophies, which manifest as hypomyelination, dysmyelination, or progressive demyelination (Naidu, 1999; Knaap and Bugiani, 2017). The protein products of genes implicated in leukodystrophies serve functions in myelin structure (*PLP1*; Tsuji, 2007), lipid metabolism (*ABCD1*; Powers et al., 2005), ion transport (*MLC1*; Knaap et al., 2012), translational regulation (*EIF2B*; Moon and Parker, 2018), and lysosomal function [*GALC* (Shin et al., 2016) and *VPS11* (Skoff et al., 2021)]. These causes highlight the complexity of myelin disorders and the variability of their clinical presentations (Gow and Lazzarini, 1996; Garbern et al., 1999).

Despite the etiological heterogeneity in myelin disorders, dysfunction or degeneration of oligodendroglia is a unifying mechanistic feature. Oligodendrocytes, responsible for myelination in the CNS, produce essential proteins for myelin production, stability and maintenance (Stecca et al., 2000) . One such protein, claudin-11, constitutes approximately 7% of total myelin protein, making it the third most abundant myelin protein after proteolipid protein 1 (PLP1) and myelin basic protein (MBP) (Bronstein et al., 1997; Harry and Toews, 1998). Recent findings identify *de novo* mutations in *CLDN11*, the gene encoding claudin-11, as a novel cause of hypomyelinating leukodystrophy (HLD), specifically leading to HLD22 (Riedhammer et al., 2020). The mechanisms by which such *de novo* mutations in claudin-11 lead to disease remain unclear. Understanding these processes is vital for developing therapies.

This review examines the biological roles of claudin-11, the importance of myelin and claudin-11 in health and disease, and the clinical features and postulated mechanisms underlying HLD22.

## 2 Molecular biology of claudin-11

**Figure 1:**
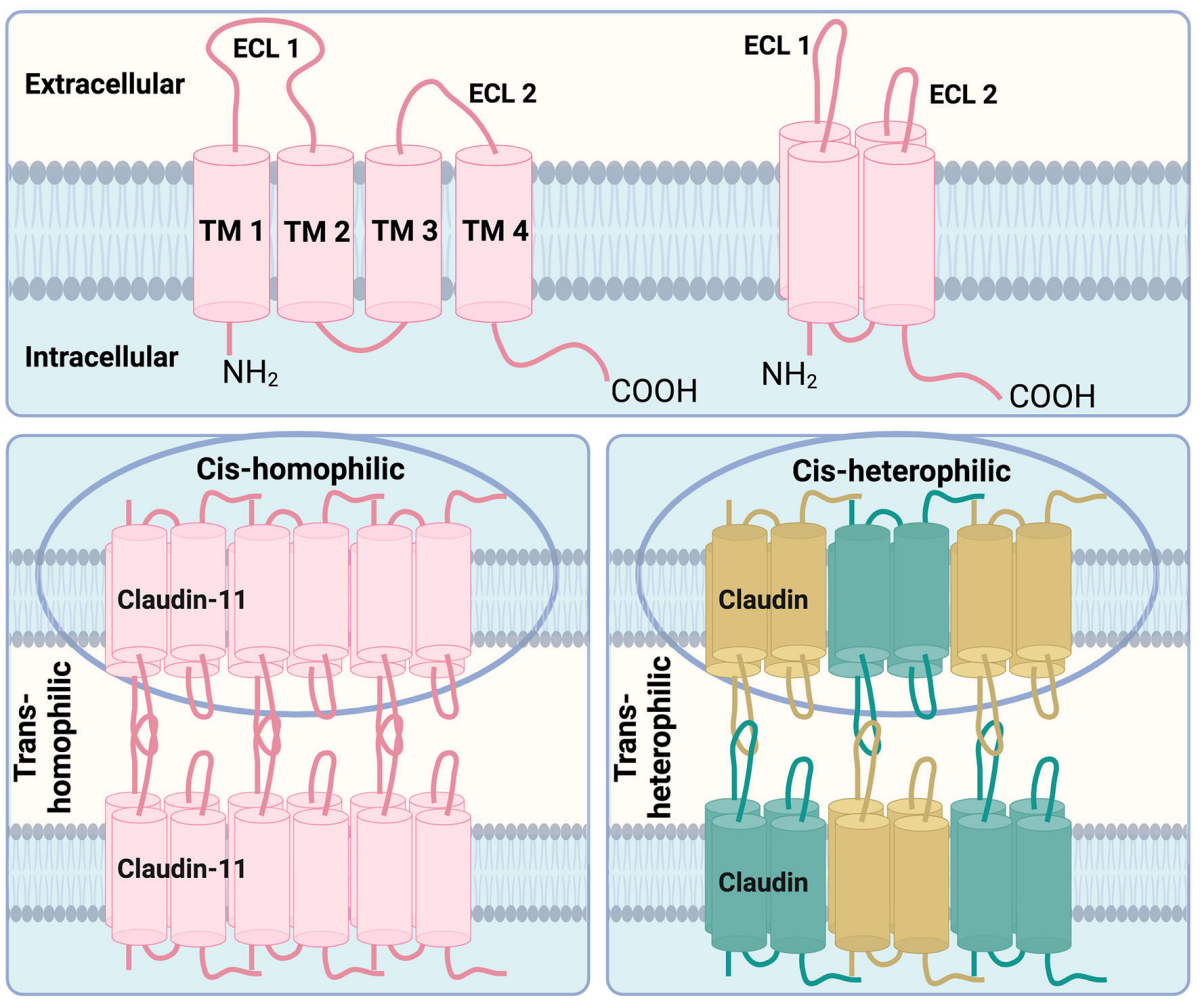
Structural features of claudin-11 tight junctions. **Top**: The key structural features of claudin family members are the four transmembrane domains (TM1, TM2, TM3, and TM4) with two extracellular loops (ECL1, ECL2) which are arranged in a 3D manner spanning across the cell membrane. **Bottom left**: Claudin-11, depicted in pink, is known to form homophilic interactions between cells (trans-homophilic) as well as homophilic interactions between claudin-11 proteins within the same membrane (cis-homophilic). **Bottom right**: Other claudin family members are known to form heterophilic interations between different claudin family proteins, shown in green and gold, within the same cell membrane (cis-heterophilic) as well as on opposing cell membranes (trans-heterophilic). Adapted from Krause et al. (2008).

The claudin family comprises 30 proteins, at least 26 of which are found in humans (Mineta et al., 2011; Engelund et al., 2012). Functionally, claudins form tight junctions (TJs) between polarized cells, regulating ion transport (Colegio et al., 2002; Koziel et al., 2020). Structurally, they are tetraspan, transmembrane proteins with sizes ranging from 20–34kDa and feature two extracellular loops critical for oligomerization and TJ function (Goncalves et al., 2013). Based on sequence homology, claudins are classified into classic and non-classic types. Non-classic claudins, to which claudin-11 belongs, share less sequence similarity to classic claudins (Krause et al., 2008; Lal-Nag and Morin, 2009). While claudins typically exhibit some overlap in their patterns of expression, claudin-11 is uniquely localized to the TJs of CNS myelin, the inner ear, and testis (Gow et al., 1999; Morita et al., 1999), with rare exceptions (Wolburg et al., 2001). Claudin proteins engage in both cis (within the same membrane) and trans (across opposing membranes) interactions, either with the same claudin (homophilic), or different claudins (heterophilic) (Krause et al., 2008). Claudin-11 is known to form homophilic interactions (Figure 1).

### 2.1 Claudin-11 in the central nervous system

**Figure 2:**
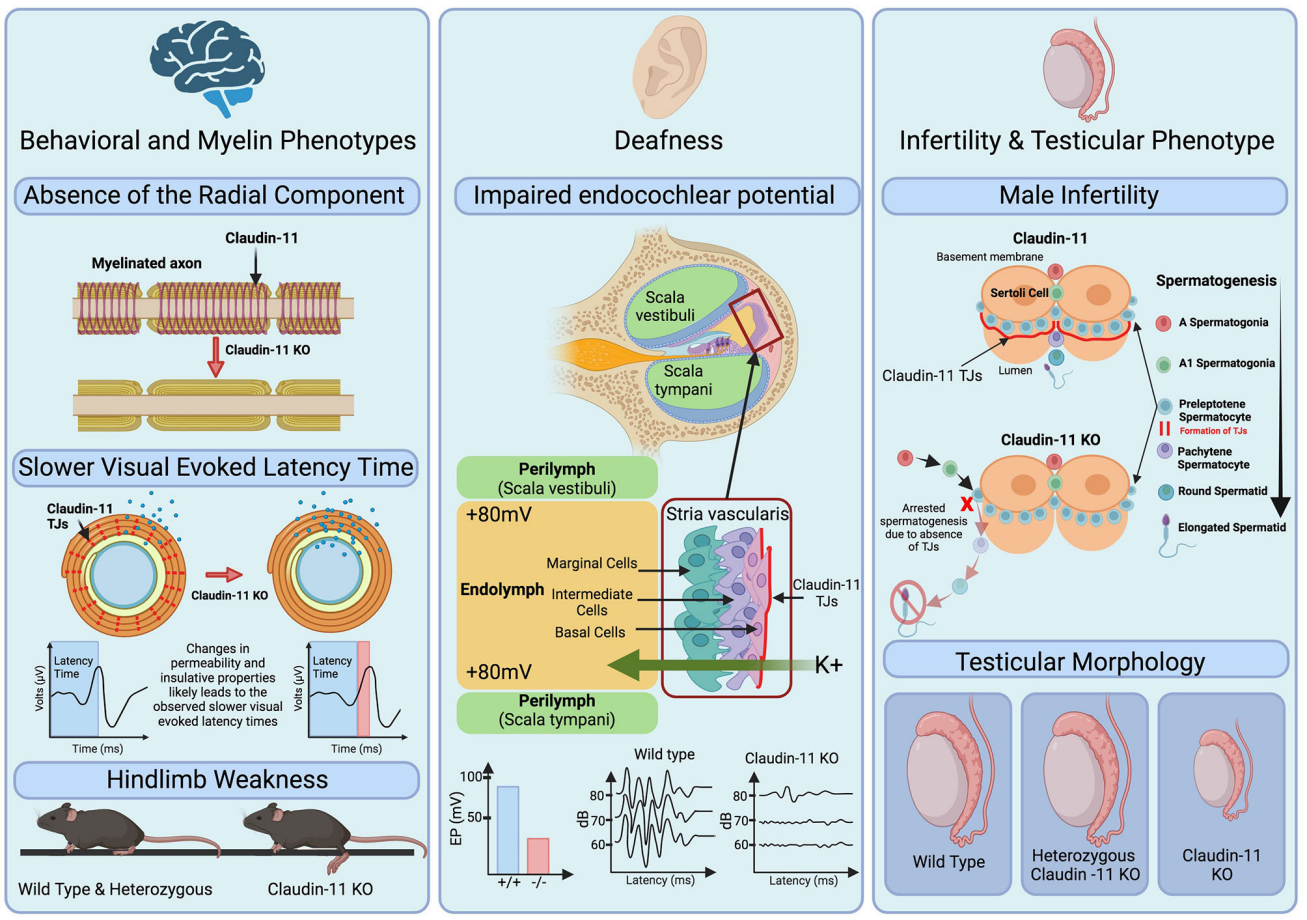
Role of claudin-11 tight junctions in myelin function, hearing, and male fertility. **Left**: Claudin-11 knock out (KO) mice demonstrate an observable absence of the radial component of the myelin sheath, depicted as the spiral. This may lead to changes in permeability and insulative properties of the myelin sheath as shown by the changes in ions (blue) present within and outside the sheath. The complete absence of claudin-11 and resulting delayed visual evoked latency time is highlighted by red in the visual evoked latency graphs. Additionally, KO mice exhibit hind limb weakness as shown in the bottom of the pannel. **Middle**: Deafness is a phenotype observed in claudin-11 KO mice. Graphs depict decreased endocochlear potential (EP) generated by KO mice and highlight the increased threshold of hearing for claudin-11 KO mice. **Right** [adapted from Smith and Braun (2012)]: Male infertility is observed in claudin-11 KO mice. During spermatogenesis, claudin-11 TJs form between sertoli cells which contributes to the blood testis barrier. In the absence of claudin-11, spermatogenesis is arrested resulting in male infertility and is accompanied by hypogonadism shown in the bottom of the pannel.

Initially identified as oligodendrocyte specific protein (OSP), claudin-11 expression in the CNS is specific to oligodendrocytes (Bronstein et al., 1996, 1997) (Figure 2). During early development, claudin-11 is expressed in mesenchymal cells adjacent to meningeal cartilage-rich areas, while postnatally, claudin-11 expression in the CNS is restricted to early oligodendrocyte progenitors and their lineage (Bronstein et al., 2000). Topologically, claudin-11 resembles other oligodendrocyte-expressed myelin proteins such as PLP1 (Gow et al., 1997; Krause et al., 2008; Devaux et al., 2010). Unlike PLP1, however, claudin-11 is localized exclusively within the radial component between the layers of the myelin sheath (Gow et al., 1999). This specific localization is critical for regulating ion diffusion and myelin permeability, which is essential for normal electrophysiological function (Devaux and Gow, 2008; Gow and Devaux, 2008; Rasband et al., 2012). Claudin-11 expression within the CNS is restricted to the oligodendrocyte lineage, however, outside of the CNS several other cell types are found to express this unique protein.

### 2.2 Claudin-11 in the inner ear

Outside of the CNS, claudin-11 is expressed in the basal cells of the stria vascularis of the inner-ear (Gow et al., 2004; Kitajiri et al., 2004a,b) and the Sertoli cells of the testes (Gow et al., 1999; Stanton, 2016) (Figure 2). The human inner ear contains thousands of mechanoreceptors essential for hearing (Liu et al., 2017). A potassium-rich endolymph compartment within the cochlea generates the endocochlear potential (EP), an electric potential crucial for transducing acoustic stimuli into electrical signals (Gow et al., 2004; Kitajiri et al., 2004b; Liu et al., 2017; Zhang et al., 2020). By regulating ion movement across the stria vascularis, claudin-11 serves a dual role in the cochlea as both conductor and isolator, effectively functioning as a “conventional electrochemical battery” for ion transport (Liu et al., 2017). Studies in knockout mouse models have revealed the indispensable role of claudin-11 in hearing. Despite having no clear gross morphological malformations within the inner ear, claudin-11 knockout mice are deaf as confirmed through auditory brainstem analysis (Gow et al., 2004; Kitajiri et al., 2004b). Furthermore, freeze fracture electron microscopy showed the absence of TJs in the stria vascularis, highlighting the critical role of claudin-11 TJs in hearing (Gow et al., 2004; Kitajiri et al., 2004b).

### 2.3 Claudin-11 in the testes

In addition to the CNS and cochlea, claudin-11 is also expressed in the testes (Gow et al., 1999) (Figure 2). Several claudin family members, including claudin-1, -3, -5, -11, -12, and -13, are present in the blood-testis barrier (Stanton, 2016). Interestingly, claudin-11 is the sole essential claudin for spermatogenesis, as its absence in knockout mice leads to infertility, whereas loss of claudin-1, -3, -12, or -13 does not affect fertility (Gow et al., 1999; Stanton, 2016). The role of claudin-5 in spermatogenesis remains unresolved due to the postnatal lethality of its knockout models (Stanton, 2016).

### 2.4 Claudin-11 in cancer

Outside normal physiology, claudin-11 expression has been implicated in several cancer types. Interestingly, both claudin-11 silencing and overexpression have been linked to invasiveness of cancer cells. For example, in gastric cancers, hypermethylation of *CLDN11* and downregulation of its expression were associated with increased cancer cell motility and invasiveness (Agarwal et al., 2009). Similar observations were reported in nasopharyngeal carcinoma cells (Li et al., 2018). Conversely, studies investigating epithelial-mesenchymal transition (EMT) mechanism implicated in cancer metastasis report increased expression of *CLDN11* correlating with less favorable patient outcomes (Li et al., 2019). These findings suggest that reduced claudin-11 expression in certain cancers, such as the gastric and nasopharyngeal types, may promote cell migration through loosening of cellular junctions (Agarwal et al., 2009; Li et al., 2018). In contrast, claudin-11 upregulation in EMT-linked cancers may promote maintenance of cell clusters, thus facilitating more efficient cancer cell dissemination (Li et al., 2019).

## 3 The role of claudin-11 in myelination

### 3.1 Expression of claudin-11 in the central nervous system

Claudin-11 is expressed in both oligodendrocyte progenitor cells (OPCs) and oligodendrocytes (OLG) (Bronstein et al., 2000). OPCs are bipolar, migratory, and proliferative cells expressing platelet-derived growth factor receptor A (PDGFRa) and neural/glial antigen 2 (NG2), which differentiate into pre-myelinating O4- and O1-positive oligodendrocytes that ultimately differentiate into non-proliferative, myelin-producing OLGs (Pouwels et al., 2014; Kuhn et al., 2019). This myelination process entails a specialized, energy-intensive membrane expansion, increasing the membrane surface area by over six thousand-fold in order to ensheath axons (Pouwels et al., 2014).

### 3.2 Claudin-11 in myelin

The specialized membrane of the myelin sheath contains many key transmembrane proteins such as PLP1 and claudin-11 which are critical to its integrity and function. Claudin-11 is unique in that it is the sole claudin family member expressed in CNS myelin and therefore its loss cannot be compensated for by other claudin proteins (Gow et al., 1999; Furuse, 2009). Studies using claudin-11 null mice support this notion. Indeed, while claudin-11 null mice, which lack the radial component, do not show gross changes in ultrastructural features of myelin such as thickness or compaction, they do exhibit slower conduction velocities across myelinated axons as well as hind limb weakness (Gow et al., 1999; Devaux and Gow, 2008; Gow and Devaux, 2008). These findings demonstrate that claudin-11 is critical for the maintenance of the electrochemical gradients and electrical resistance properties within the myelin sheath (Gow et al., 1999; Devaux and Gow, 2008; Gow and Devaux, 2008).

### 3.3 Oligodendrocyte migration

Outside its role in the radial component of myelin, claudin-11 has been linked to OLG proliferation and migration. Using a yeast two-hybrid system, claudin-11 was found to interact and form a complex with OSP/claudin-11-associated protein (OAP-1) and beta-1 integrin (Tiwari-Woodruff et al., 2001). Overexpression of claudin-11 and OAP-1 increased proliferation in a conditionally immortalized mouse oligodendrocyte (CIMO) cell line (Bronstein et al., 1998; Tiwari-Woodruff et al., 2001). Antibodies targeting claudin-11, OAP-1, and beta-1 integrin led to a marked reduction in CIMO cell migration (Tiwari-Woodruff et al., 2001). These findings suggest that the claudin-11/OAP-1/beta-1 integrin complex may be involved in the regulation of migratory and proliferative behavior of OLGs (Tiwari-Woodruff et al., 2001). Despite these *in vitro* observations, overt impairment or abnormal proliferation and migration of OLGs have not been reported in claudin-11 null mice (Devaux et al., 2010).

## 4 *CLDN11*-related hypomyelinating leukodystrophy

### 4.1 Definition of hypomyelinating leukodsytrophy

White matter diseases are classified into subcategories based on the predominant nature of the myelin pathology observed and include demyelinating, myelinolytic, and dysmyelinating conditions (Naidu, 1999; Knaap and Bugiani, 2017). Myelinolytic diseases, exemplified by Canavan disease, are characterized by unique vacuolar myelinopathy (Naidu, 1999). Demyelinating conditions, such as multiple sclerosis, involve destruction of biochemically normal myelin (Naidu, 1999). Dysmyelinating diseases, notably leukodystrophies, often have genetic causes affecting myelin formation or function (Naidu, 1999). The term “leukodystrophy,” derived from “leuko” meaning white and “dystrophy” meaning wasting, is a fitting descriptor highlighting the degeneration and atrophy of CNS white matter observed in these conditions (Vanderver et al., 2015). The first documented leukodystrophy was described in 1885 and 1910 by Pelizeaus and Merzbacher, identifying a familial progressive white matter disorder known as PMD (Koeppen and Robitaille, 2002). HLDs, such as PMD and HLD22, are progressive disorders (Naidu, 1999; Knaap and Bugiani, 2017). Clinically, they manifest as developmental delay, ataxia, spasticity, hypotonia, and varying degrees of intellectual disability (Pouwels et al., 2014; Knaap and Bugiani, 2017). A defining feature of HLDs is the abnormal development or deposition of myelin (Pouwels et al., 2014). This is typically accompanied by variety of pathological alterations, such as neuronal loss, dysmyelination, demyelination, and axonal damage, that manifest to varying degrees depending on the underlying genetic defect (Sima et al., 2009; Gruenenfelder et al., 2020). HLDs are typically diagnosed through magnetic resonance imaging (MRI) manifesting as lower T2 hypointensities without much reduction in T1 hyperintensities (the latter is typically seen in other non-HLD leukodystrophies) (Pouwels et al., 2014).

### 4.2 Hypomyelinating leukodystrophy 22

*CLDN11* was identified as a novel cause of HLD in 2020 through trio exome sequencing in three unrelated pediatric patients (2 male, 1 female) (Riedhammer et al., 2020). These patients carried *de novo*, heterozygous stop-loss variants: two had a c.622T>C, p.(*208Glnext*39) variant, while the third patient had the c.622T>G, p.(*208Gluext*39) variant (Riedhammer et al., 2020). Although additional disease-associated variants are listed in databases such as ClinVar and gnomAD, with associated phenotypes like marfanoid habitus and intellectual disability (National Center for Biotechnology Information, 2022), only the stop-loss variants are categorized as pathogenic to date.

The *de novo* stop-loss variants are predicted to result in a 39-amino acid extension at the cytoplasmic C-terminal end of claudin-11 (Riedhammer et al., 2020). This extension is predicted to form an alpha helix that does not integrate into the cytoplasmic membrane (Riedhammer et al., 2020). Imaging findings of the patients showed that T1-weighted images had signal intensity near normal, whereas T2-weighted images showed reduced myelination of white matter structures (Riedhammer et al., 2020). All three individuals presented with delayed developmental milestones, contractures, nystagmus, drooling, hypermetropia, severe disarticulation, and require mobility support when walking and climbing stairs, features that are shared with PMD and other HLDs (Riedhammer et al., 2020). Interestingly, hypermetropia is not a common clinical feature of HLD, suggesting its potential as a diagnostic marker for HLD22 (Riedhammer et al., 2020). HLD22 affects paediatric patients and is both early onset and progressive (Riedhammer et al., 2020). The underlying mechanisms for this disease are currently not well understood, and effective treatments are currently lacking.

Since the description of the first three patients with *CLDN11*-related HLD22, at least three more individuals have been identified with a clinical phenotype compatible with HLD22 and with *CLDN11* variants leading to an extended protein (unpublished). Currently, it is not clear whether *CLDN11* variants causing other types of changes in the protein are associated with a similar phenotype in humans.

### 4.3 Cellular mechanisms of claudin-11 dysfunction in hypomyelinating leukodystrophy 22

#### 4.3.1 Mouse models of claudin-11 deficiency

Mouse models, particularly the *Cldn11* null and heterozygous knockouts, have provided key insights into the role of claudin-11 in myelination. Behavioral phenotyping indicates reduced anxiety in the *Cldn11* null mice from an early age as assessed using open field and the marble burying tests (Maheras et al., 2018). These anxiety manifestations precede age-dependent, site-specific alterations in inhibitory and excitatory neurotransmitters, and have instead been suggested to reflect a temporal disconnection in signal transmission via myelinated axons that represent a potential mechanism of neuropsychiatric disease (Maheras et al., 2018). Motor assessments revealed hindlimb weakness in the *Cldn11* null animals, although heterozygous mice were phenotypically similar to wild type controls (Gow et al., 1999). The absence of neurodegeneration markers such as N-acetylaspartate or spinal pathology suggests the behavioral changes may arise from delayed signal transduction by myelinated axons rather than axonal degeneration or neuronal loss (Maheras et al., 2018). This notion is corroborated by visual evoked potential assessments, auditory brain stem evoked potentials, and compound action potentials in the optic nerve and spinal cord showing slowed latency times in the *Cldn11* null mouse model (Gow et al., 1999, 2004; Gow and Devaux, 2008). These studies showcase the utility of mouse models in understanding claudin-11 biology, and suggest that similar models may aid in gaining insights into the pathogenic mechanisms underlying HLD22 as well as evaluating potential therapeutics for the disease.

Myelin specific analysis of the *Cldn11* null mouse model revealed the absence of radial tight junctions typically present in myelin, though other ultrastructural features are preserved (Gow et al., 1999). To date, no deficits in oligodendrocyte proliferation and migration have been reported in these mice (Devaux et al., 2010). Nonetheless, a comprehensive analysis of the cellular composition of the CNS of *Cldn11* null mice is lacking. A detailed analysis would be required to definitively conclude that different cellular populations, including oligodendrocytes, are not affected in these mice.

In addition to myelin-related alterations, *Cldn11* null mice present with two other distinct phenotypes: deafness and sterility. The absence of claudin-11 compromises endocochlear potential and mice present with deafness (Gow et al., 2004; Kitajiri et al., 2004b). The infertility is restricted to males; *Cldn11* null females are able to produce litter sizes comparable to controls (Gow et al., 1999). Male infertility in *Cldn11* null mice is associated with significantly smaller testis (Gow et al., 1999) and is likely due to arrested spermatogenesis (Plymate, 1994; Wu et al., 2012). Notably, this is another instance where the heterozygous mice are indistinguishable from controls, demonstrating that one functional copy of *Cldn11* is sufficient for normal physiological function (Gow et al., 1999). These findings raise questions about the pathogenic mechanism(s) of HLD22, as they collectively indicate that haploinsufficiency is unlikely to cause disease.

#### 4.3.2 Postulated mechanisms of mutant claudin-11-mediated HLD22

The endoplasmic reticulum (ER) is crucial for protein folding and post-translational modifications (Helenius et al., 1992). Given the massive scale of protein production required for myelination in oligodendrocytes (Anitei and Pfeiffer, 2006) increased ER stress has been implicated in several myelinopathies (Southwood et al., 2002; Voorn et al., 2005; Mhaille et al., 2008; Pennuto et al., 2008) [Reviewed in Lin and Popko (2009)]. The unfolded protein response (UPR) is activated when there is an accumulation of unfolded proteins within the ER (Read and Schroder, 2021). The UPR consists of three signalling pathways: the IRE1 pathway is triggered to enhance protein folding and degradation (Adams et al., 2019); the PERK pathway phosphorylates eukaryotic translation initiation factor 2α (EIF2α), reducing protein translation (Harding et al., 1999; Adams et al., 2019); and the ATF6 pathway, similar to PERK, boosts protein folding and degradation (Yoshida et al., 2001; Adams et al., 2019). Unresolved protein misfolding, despite sustained activation of these UPR pathways, leads to apoptosis of oligodendrocytes (Gow et al., 1998).

Increased ER stress and UPR activation have been implicated in PMD as a result of mutations or altered expression of PLP1, a tetraspan, secretory pathway myelin protein (Southwood et al., 2002). As claudin-11 is also a secretory pathway, tetraspan myelin protein, a similar pathogenic mechanism may underlie HLD22. Specifically, mutations in claudin-11 may lead to protein misfolding and accumulation in the ER, activation of the UPR, and ultimately oligodendrocyte death and hypomyelination. It is also possible that other gain-of-toxic function mechanisms involving aberrant claudin-11 localization or interactions may contribute to HLD22. Given that one functional claudin-11 copy suffices for normal physiology as shown in animal models, a claudin-11 loss-of-function scenario appears unlikely as the cause of HLD22, though dominant negative effects cannot be ruled out.

## 5 Conclusion

Claudin-11 plays a critical role in maintaining several aspects of normal physiology, including forming the radial component of the myelin sheath and facilitating nerve conduction, maintenance of the endocochlear potential required for normal hearing, and maintaining the integrity of the blood-testis barrier necessary for normal spermatogenesis. Abnormal claudin-11 function has been implicated in various pathologies ranging from cancer to behavioral and myelin abnormalities, and most notably the hypomyelinating disorder HLD22. Mouse and *in vitro* models serve as important tools to elucidate the disease characteristics and pathogenic mechanisms associated with claudin-11. A comprehensive understanding of the biological function of claudin-11 and the pathogenic mechanisms underlying HLD22 is essential for the development of effective treatments for this disease.

## Author contributions

SG: Conceptualization, Data curation, Project administration, Visualization, Writing—original draft, Writing—review & editing. OO: Writing—review & editing. AG: Writing—review & editing. SS-I: Writing—review & editing. MP: Conceptualization, Funding acquisition, Project administration, Resources, Supervision, Writing—review & editing, Visualization.
